# Adaptability to Balance Perturbations During Walking as a Potential Marker of Falls History in Older Adults

**DOI:** 10.3389/fspor.2021.682861

**Published:** 2021-05-19

**Authors:** Marissa H. G. Gerards, Kenneth Meijer, Kiros Karamanidis, Lotte Grevendonk, Joris Hoeks, Antoine F. Lenssen, Christopher McCrum

**Affiliations:** ^1^Department of Epidemiology, Care and Public Health Research Institute, Maastricht University, Maastricht, Netherlands; ^2^Department of Physical Therapy, Maastricht University Medical Center+, Maastricht, Netherlands; ^3^Department of Nutrition and Movement Sciences, NUTRIM School of Nutrition and Translational Research in Metabolism, Maastricht University, Maastricht, Netherlands; ^4^Sports and Exercise Science Research Centre, School of Applied Sciences, London South Bank University, London, United Kingdom; ^5^TI Food and Nutrition, Wageningen, Netherlands

**Keywords:** accidental falls, risk assessment, perturbation, aging, adaptation, stability recovery, falls prevention

## Abstract

Given that falls most commonly occur during walking due to unexpected balance perturbations like trips and slips, walking-based balance assessment including walking stability and adaptability to such perturbations could be beneficial for fall risk assessment in older adults. This cross-sectional study reanalyzed data from two larger studies conducted with the same walking protocol. Participants completed unperturbed walking trials at speeds of 0.4 m/s up to 1.8 m/s in 0.2 m/s steps. Ten unannounced treadmill belt acceleration perturbations were then applied while participants walked at equivalent stability, assessed using the margins of stability. Retrospective (12 months) falls incidence was collected to divide participants into people with and without a history of falls. Twenty older adults (mean age 70.2 ± 2.9 years) were included in this analysis; eight people with one or more recent falls and 12 people without, closely matched by sex, age and height. No significant differences were found in unperturbed walking parameters or their variability. Overall perturbation-recovery step behavior differed slightly (not statistically significant) between the groups after the first perturbation and differences became more pronounced and significant after repetition of perturbations. The No-Falls group significantly reduced the number of recovery steps needed across the trials, whereas the Falls group did not show these improvements. People with a previous fall tended to have slightly delayed and more variable recovery responses after perturbation compared to non-fallers. Non-fallers demonstrate more signs of adaptability to repeated perturbations. Adaptability may give a broader indication of the ability of the locomotor system to respond and improve responses to sudden walking perturbations than unperturbed walking variability or recovery to a single novel perturbation. Adaptability may thus be a more useful marker of falls history in older adults and should be considered in further research.

## Background

Falls are a principal cause of injury, leading to disability and hospitalization in older adults (Berry and Miller, 2008). Therefore, adequate identification and treatment of older fallers are critical. Approximately 60% of outdoor falls in older adults occur when unexpected balance perturbations during walking (e.g., slips or trips) cause a sudden change in the relationship between the center of mass (CoM) and base of support (BoS) of the body (Berg et al., 1997). Thus, balance assessment during walking, focusing on walking stability and adaptability may be beneficial for fall risk assessment in older adults (Woollacott and Tang, 1997; Pai et al., 2010a; McCrum, 2020a).

In response to balance perturbations such as slips and trips, older adults show less effective initial recovery responses than younger adults (Pijnappels et al., 2005; Karamanidis and Arampatzis, 2007; Pai et al., 2010b). Still, the literature reports that older adults seem fully capable of improving their responses when exposed to repeated perturbations (Pai et al., 2014; Bohm et al., 2015; McCrum et al., 2017). As a result, walking stability in response to single and repeated perturbations may capture different underlying mechanisms. However, how adaptability to repeated perturbations relates to real life falls has not been the topic of many studies. Pai et al. (2010a) associated adaptability to repeated slip perturbations during a sit-to-stand task with a lower likelihood of future falls in daily life in older adults. Adaptability was indicated by less balance loss and falls during the task and improved recovery performance during the final slip. This association has not yet been thoroughly investigated for mechanical perturbations during walking, which are more task-specific to the most common causes of falls in older adults.

In this study, we aim to address the extent to which stability following a single perturbation and adaptability following repeated perturbations relate to falls history in older adults. Stability of the body configuration during walking will be measured using the margin of stability (MoS) (Hof et al., 2005). Due to previous indications of differences between older adults with and without a history of falls (Hausdorff et al., 2001; Mortaza et al., 2014) we also analyze step variability during unperturbed walking, to examine how these potential differences relate to those seen in the perturbation tasks. These analyses may give indications of the usefulness of such tasks and properties for falls risk assessments and falls prevention. We hypothesize that there will be not only higher step variability during walking, but also a reduced ability to cope with and adapt to unexpected balance perturbations during walking in older adults who fell in the past 12 months compared to older adults who did not fall.

## Methods

### Setting and Subjects

This cross-sectional study reanalyzed data from two larger studies that included the same walking protocol (McCrum et al., 2020; Grevendonk et al. submitted). Older adults were recruited from the city of Maastricht, the Netherlands, and the surrounding area. Inclusion criteria were; community-dwelling, 65–80 years old, no known musculoskeletal or neurological deficits and no history of dizziness, balance or walking complaints. All subjects provided written informed consent. Both studies were approved by the medical ethics committee (METC) at Maastricht University Medical Centre (MUMC+) (NL58205.068.16 & NL59895.069.17) and were conducted in accordance with the declaration of Helsinki. Prior to the walking measurements, participants were given a short falls history questionnaire based on the recommendations of Lamb et al. (2005) and Lord et al. (2011), that led with the question: “In the past year, have you had any fall including a slip or trip in which you lost your balance and landed on the floor or ground or lower level?” This was followed by other questions about the number, location and cause of the fall(s) and about any injuries sustained. The questionnaire is available from https://osf.io/hmjef/ (McCrum, 2020b). Participants were divided into two groups based on their answers to this questionnaire. The Falls group including those participants who reported one or more falls in the past year, and the No-Falls group including those who did not fall.

For the current secondary analysis, a sample size calculation was conducted to determine the required sample size for α = 0.05, β = 0.8 and estimated effect size of *f* = 0.5 for the group effect (falls history vs. no falls history) on MoS in a two-way ANOVA, with step as the other (repeated measures) factor (Baseline, pre-perturbation and the first eight recovery steps). This effect size for the MoS across the steps corresponds to a Cohen's *d* of 1 and to an approximately three-step difference in recovery to baseline MoS based on previous analyses (McCrum et al., 2020), which we interpret to be clinically meaningful. This revealed a required total sample of 20 participants. All available fallers from the existing datasets were included in the reanalysis, and a group of non-fallers was formed from participants who most closely matched the fallers in sex, age, and height.

### Setup

Measurements were conducted with the Computer Assisted Rehabilitation Environment Extended (CAREN; Motekforce Link, Amsterdam). This comprises of a dual-belt force plate-instrumented treadmill (1,000 Hz), a 12 camera Vicon Nexus motion capture system (100 Hz; Vicon Motion Systems, Oxford, UK) and a 180° virtual environment providing optic flow. A safety harness connected to an overhead frame was worn by the participants. Six retroflective markers were attached to anatomical landmarks (C7, sacrum, left and right trochanter and left and right hallux) to calculate MoS.

### Procedures

**Figure 1:**
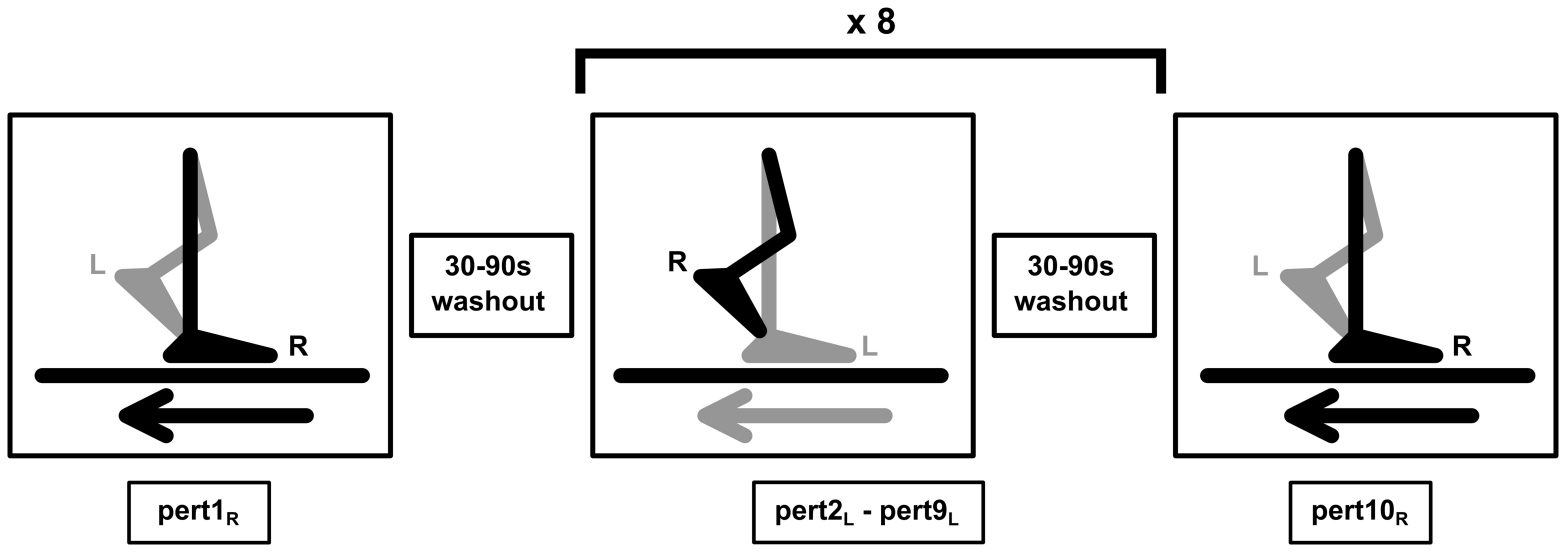
Gait perturbation protocol [image previously shown in McCrum et al. (2019b)]. The right leg (R) was perturbed by the treadmill belt acceleration first (Pert1_R_), followed by eight perturbations (Pert2_L_ – Pert9_L_) to the left leg (L), and the final perturbation (Pert10_R_) was again applied to the right leg (R). In all, 30–90 s of unperturbed walking occurred between each perturbation. The perturbation was designed to cause a forward rotation and acceleration of the upper body, relative to the lower body, leading to a forward loss of dynamic stability.

Participants completed familiarization trials followed by measurement trials from speeds of 0.4 m/s up to 1.8 m/s in 0.2 m/s steps. To ensure equivalent stability across participants and groups during the perturbation trials, the stability-normalized walking speed was then calculated using the mean anteroposterior MoS of the final 10 steps of each walking trial [(0.4–1.8 m/s) (McCrum et al., 2019b)]. The method and effectiveness of this approach are described in detail elsewhere (McCrum et al., 2019b). For each participant, the walking speed that would result in MoS of 0.05 m was calculated. The walking perturbation protocol then began with participants walking at the stability-normalized speed for 3-4 min, followed by 10 unilateral treadmill belt acceleration perturbations, which occurred unannounced every 30–90 s. The perturbation was a 3 m/s^2^ acceleration of the treadmill belt to a maximum speed equal to 180% of the stability-normalized walking speed. The acceleration began when the hallux marker of the to-be-perturbed limb passed the hallux marker of the opposite foot in the sagittal plane. The belt decelerated at toe-off of the perturbed limb. Participants were naïve to the specifics of the perturbation protocol (i.e., limb, type, number, timing, magnitude). The first and tenth accelerations perturbed the right leg, while the second to ninth accelerations perturbed the left leg. This way, not only balance recovery after a novel perturbation, but also adaptation to repeated perturbations can be studied within the same protocol. A schematic overview of the perturbation protocol is shown in Figure 1. Further technical details of the perturbations can be found elsewhere (McCrum et al., 2018).

### Data Processing

Data processing was conducted in MATLAB (2016a, The MathWorks, Inc., Natick). The three-dimensional coordinates of the markers were filtered using a low pass second order Butterworth filter (zero-phase) with a 12 Hz cut-off frequency. Foot touchdown and toe-off were detected using marker and force plate data, as described previously (McCrum et al., 2019a). The anteroposterior MoS at foot touchdown were calculated as the anteroposterior distance between the anterior boundary of the base of support (BoS) and the extrapolated center of mass, adapted for our validated reduced kinematic model (Hof et al., 2005; Süptitz et al., 2013). The MoS was calculated for the following steps: baseline for each perturbation was the mean MoS of the eleventh to second last step before each perturbation (Base); the final step before each perturbation (Pre); and the first eight recovery steps following each perturbation (Post1-8). The number of steps to return to baseline stability following the perturbation was determined by calculating the number of steps that were within 0.05 m of the MoS value of Base for each individual, counting back from the eighth recovery step, using custom written R code (R version 3.6.0; R Core Team, 2019). Additionally, the means and coefficients of variation of step length, width and time, as well as double support time, were calculated using the foot marker data for 0.4, 0.8, 1.2, and 1.6 m/s unperturbed walking trials.

### Analysis

The effects of falls history on MoS recovery after the first perturbation to each leg (Pert1_R_ and Pert2_L_; representing the un-adapted response) and the final perturbation to the left leg (Pert9_L_; representing the adapted response), were analyzed using repeated-measures two-way ANOVA with group (Falls/No-Falls) and step (repeated measures: Base, Pre, Post1-8) as factors for each of the perturbations separately. Additionally, Mann-Whitney tests were applied to compare the groups on number of recovery steps needed for each perturbation and Friedman tests were used to assess the change in steps across perturbations within each group. Finally, the spatial (step length and width means and variability) and temporal (step and double support time means and variability) parameters of gait at a range of walking speeds (0.4, 0.8, 1.2, and 1.6 m/s) were compared between the Falls and No-Falls groups using a two-way ANOVA with group (Falls/No-Falls) and walking speed (repeated measure) as factors.

## Results

Twenty older adults (8 with, and 12 without falls in the previous year) were included in this study. Characteristics of participants described by group (Falls/No-Falls) can be found in Table 1: participant characteristics. Six of the eight participants in the Falls group fell only once in the previous year, one reported two falls, and one fell three or more times.

**Table 1:** Participant characteristics (mean ± SD).

	**Falls group**	**No-falls group**
Men/women (*n*)	4/4	6/6
Age (years)	70.6 ± 3.6	70 ± 2.4
Height (cm)	168.2 ± 15.4	169.4 ± 7.2
Weight (kg)	75 ± 16.3	75.6 ± 10.3
Body mass index	26.3 ± 3.3	26.3 ± 2.9
Stability-normalized walking speed (m/s)	1.29 ± 0.13	1.31 ± 0.14
Falls in the previous year n (frequency)	1 (6), 2 (1), ≥3 (1)	0 (12)

### Step Parameters

Spatial and temporal parameters of gait, as well as their variability, were compared between groups using two-way repeated-measures ANOVAs. From these analyses, no significant effects of group (Falls vs. No-Falls), and no interaction effects (Group x Speed) were found for any parameter (the complete effect and interaction results can be found in Supplementary Table 1).

### Stability and Adaptability

**Figure 2:**
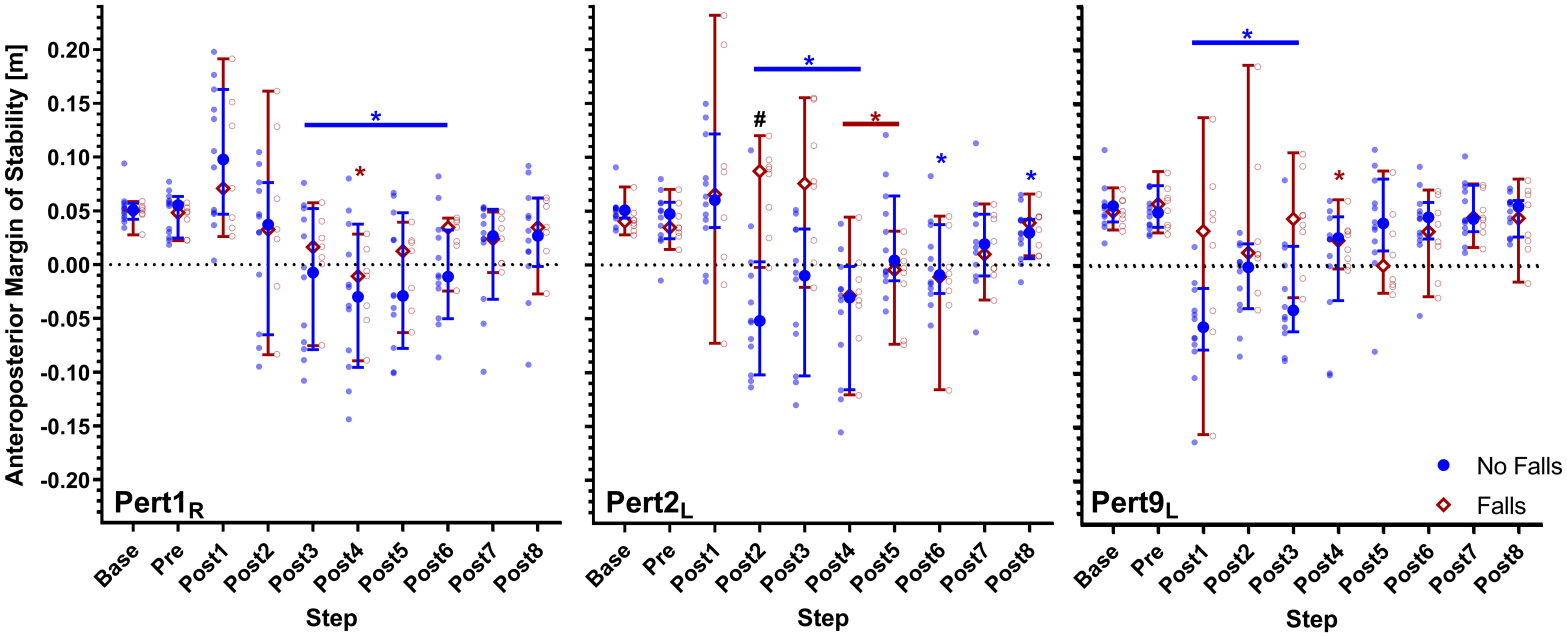
Median and 95% confidence intervals (with individual data points) of the anteroposterior margins of stability during the first, second and ninth perturbations (Pert1_R_, Pert2_L_, and Pert9_L_, respectively) including unperturbed walking prior to each perturbation (Base), the final step prior to each perturbation (Pre) and the first eight recovery steps following the perturbations (Post1–8) for Falls and No-Falls groups. Blue * and Red *: significant difference to Base for the No Falls and Falls groups, respectively (*P* < 0.05; adjusted using Dunnett's multiple comparisons test). ^#^Significant difference between the No Falls and Falls groups (*P* < 0.05; adjusted using Sidak's multiple comparisons test).

All participants were able to recover from the walking perturbations without harness assistance. However, due to a technical failure during the first perturbation, one participant was excluded from the analyses involving Pert1_R_. Two-way repeated-measures ANOVAs for Pert1_R_, Pert2_L_ and Pert9_L_ did not reveal significant effects of falls history on MoS [Pert1_R_: *F*_(1, 17)_ = 0.89, *P* = 0.36; Pert2_L_: *F*_(1, 18)_ = 3.07, *P* = 0.097; Pert9_L_: *F*_(1, 18)_ = 3.3, *P* = 0.085). Significant step by falls history interaction effects on MoS were found for Pert2_L_ and Pert9_L_ (Pert1_R_: *F*_(9, 153)_ = 0.31, *P* = 0.97; Pert2_L_: *F*_(9, 162)_ = 5.25, *P* < 0.0001; Pert9_L_: *F*_(9, 162)_ = 3.63, *P* = 0.0004). Dunnett's tests for multiple comparisons were used to compare the MoS for each step to the Base value (results indicated in Figure 1). Sidak's tests for multiple comparisons were used to compare the MoS between groups and revealed that only Post2 in Pert2_L_ was significantly different (Figure 2; note that the study was not powered for these pairwise comparisons). Complete Dunnett and Sidak results can be found in the Supplementary Material.

**Figure 3:**
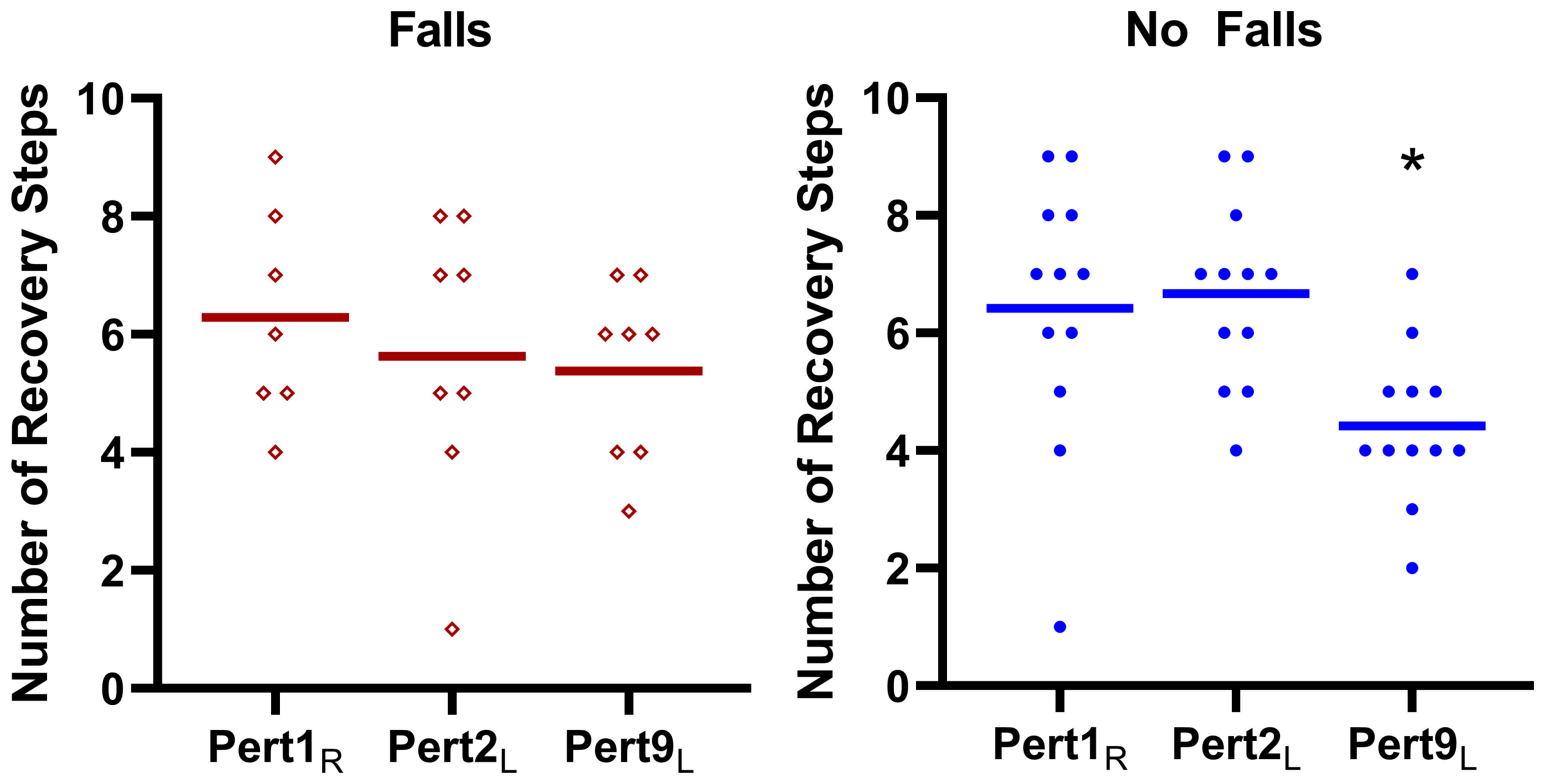
The number of recovery steps (means and individual values) required by the Falls group (left panel) and No Falls group (right panel) for the first, second and ninth perturbations (Pert1_R_, Pert2_L_, and Pert9_L_, respectively). *Significant difference to Pert1_R_ and Pert2_L_.

The Falls group required averages of 6.3, 5.6, and 5.4 recovery steps and the No Falls group required averages of 6.4, 6.6, and 4.4 recovery steps for Pert1_R_, Pert2_L_, and Pert9_L_, respectively (see Figure 3). Mann-Whitney tests did not find significant group differences in number of recovery steps (*U* = 37, *P* = 0.7; *U* = 37.5, *P* = 0.44; *U* = 31, *P* = 0.19). A Friedman test revealed a significant effect of perturbation number on the number of recovery steps in the No Falls group (Friedman statistic = 12.41, *P* = 0.002), with Dunnett's multiple comparisons tests revealing significant differences between Pert9_L_ and both Pert1_R_ (*P* = 0.018) and Pert2_L_ (*P* = 0.007). Due to the missing participant in the Falls group at Pert1_R_, Wilcoxon signed rank tests were used for this group and did not reveal significant differences in the number of recovery steps needed between Pert1_R_ and Pert2_L_ (*P* = 0.25), Pert1_R_ and Pert9_L_ (*P* = 0.53) and Pert2_L_ and Pert9_L_ (*P* > 0.99).

## Discussion

The aim of this study was to address the extent to which walking stability following a single perturbation and walking adaptability following repeated perturbations relate to falls history in older adults. We hypothesized that older adults with a history of falls would demonstrate decreased stability and adaptability compared to older adults without a history of falls. Additionally, we analyzed step variability during unperturbed walking, due to previous indications of increased variability in older adults with a history of falls (Mortaza et al., 2014).

Previous studies indicate differences in variability during unperturbed walking between older adults with and without a history of falls [for a review see Mortaza et al. (2014)]. However, in this study, no significant between-group differences in variability during unperturbed walking were found. Our study used set walking speeds instead of self-selected walking speeds, which may have resulted in differences compared to previous studies [7 out of the 13 studies reviewed in Mortaza et al. (2014) that found significant differences between fallers and non-fallers do not mention accounting for walking speed]. The results of this study (Supplementary Table 1) show significant walking speed effects on nearly all parameters, but no significant group effects.

Our results showed no significant effects of falls history on MoS during the first left or right leg perturbations (Pert1_R_ and Pert2_L_). However, significant step by falls history interaction effects on MoS were found for Pert2_L_, with a significant between-group difference in the second recovery step. The middle panel in Figure 2 shows that the No-Falls group had negative MoS on the second recovery step, while the Falls group still had positive MoS. This may be due to a difference in the recovery response directly after the perturbation, in which the Falls group shows a slightly delayed recovery compared to the No-Falls group. These differences are less pronounced but consistent with findings from another study (McCrum et al., 2020), which compared reactive stability between healthy young and older adults using the same walking perturbation protocol. In that study, older adults had a more posterior extrapolated center of mass in response to the perturbation, resulting in initially more positive MoS but a delayed stability recovery. Additionally, notably greater inconsistency in perturbation recovery responses across the Falls group compared to the No-Falls group can be observed in Figure 2, indicating there may be inconsistent recovery strategies in older adults with a history of falls. Despite more inconsistency however, the highest MoS value in the first recovery steps consistently belongs to participants in the Falls group. Combined, these results might hint at a decreased ability to coordinate the dual tasks of maintaining stability and continuing walking on the treadmill with age, and a further decrease in older adults with a history of falls compared with older non-fallers. This is consistent with findings from a study by dos Santos et al. which suggested a tendency for older fallers to favor a “stability-first” strategy, when facing other motor dual-tasks (Dos Santos et al., 2018). In their study, older fallers showed similar walking stability but decreased accuracy when placing a dowel over a target compared to non-fallers. The differences between the Falls and No-Falls groups after the first perturbation found in this study, are insufficiently pronounced to be a useful indicator of falls risk. However, corroborated with the presented literature, they suggest that the ability to coordinate a physical dual-task (combined stability recovery after a walking perturbation and continued treadmill walking) may be related to fall risk in older adults. To clarify this relationship and how it relates to daily-life situations of older adults, future studies may focus on the ability to coordinate various dual-tasks with stability recovery from perturbations during overground walking.

While the results showed no significant group effect, a significant step by falls history interaction on MoS was found for the last left leg perturbation (Pert9_L_). This indicates a difference between the groups for specific steps after this perturbation. Additionally, high variation in MoS after Pert9_L_ in the Falls group is observed (indicated by the wider confidence intervals and individual data points), as there was during the early perturbations, and the presence of some high MoS values in the first recovery steps remains. In contrast, the variability in MoS in the No-Falls group has visibly decreased by Pert9_L_, and there are no longer any high MoS values in this group in the first few recovery steps. Together, this indicates better adaptation in the No-Falls group, who by Pert9_L_, seem to respond with more consistent and effective recovery responses. Statistically this is substantiated by the significant differences in the number of recovery steps needed to reach close to normal stability values between perturbation 9 and the first two perturbations in the No-Falls group, with no significant differences in the Falls group. These findings are in alignment with results from a study by Pai et al. who demonstrated that adaptability to repeated perturbations during a sit-to-stand task may give an indication of falls risk (Pai et al., 2010a). These findings suggest that with further research, adaptation to repeated walking perturbations may be a useful measure to distinguish between older adults with and without a history of falls.

We hypothesize that recovery to a single novel treadmill acceleration perturbation is too specific a task to assess overall fall risk. The task-specificity of balance is now well established (Patla et al., 1990; Kiss et al., 2018; Ringhof and Stein, 2018) and given that falls can occur in a multitude of ways, this one specific perturbation might not represent or generalize to all possible causes of falls. Reduced adaptability, however, may give a broader indication of the ability of the locomotor system to respond and improve reactive responses to sudden perturbations, which may better generalize to the many situations that could lead to falls. It may also serve as a marker for the health of the locomotor control system (which may, in turn, be linked with falls risk), as reduced adaptability to such perturbations has often been shown in sensory and neurological pathology (Karamanidis et al., 2020). How the proposed relation between adaptability to repeated perturbations, locomotor system health and falls risk presents in daily-life remains unclear, and should be studied further. Additionally, there are many ways that walking adaptability can be assessed, and it is currently unclear if the method of assessment is critical (Geerse et al., 2019). Further research on walking adaptability in various tasks, including repeated external perturbations such as slips or trips, in older fallers and non-fallers, could help address this gap in knowledge.

We included a relatively healthy sample of older adults, resulting in mostly older adults who had experienced a single fall in the Falls group (with no known musculoskeletal or neurological deficits and no history of dizziness, balance or walking complaints), which may decrease the generalizability of the results to more frail populations. However, it is in this relatively healthy part of the older population where other clinical tests are known to have ceiling effects, which makes it important to determine other methods of indicating increased risk of falls for this population (Petterson et al., 2020). Having experienced one or more previous falls is one of the strongest predictors for future falls in community-dwelling older adults (OR 2.8 for all fallers; OR 3.5 for recurrent fallers) (Deandrea et al., 2010).

In conclusion, this study found some small but significant differences in reactive stability and adaptability between older adults with and without a history of falls, but no differences in variability of unperturbed walking. The results indicate that older adults with a history of falls may have decreased ability to coordinate the dual tasks of regaining stability and continuing to walk on the treadmill. The differences between the groups were more pronounced after repeated perturbations, with evidence of better adaptation in the No-Falls group, while increased variability of recovery responses and signs of a different recovery strategy remained in the Falls group. The results from the present study indicate that further research on adaptability to repeated walking perturbations as an indicator of falls history, and how this presents in the daily life of older adults, is warranted.

## Data Availability Statement

The original contributions presented in the study are included in the article/Supplementary Material, further inquiries can be directed to the Corresponding author.

## Ethics Statement

The studies involving human participants were reviewed and approved by Medisch Ethische Toetsingscommissie azM/UM, Maastricht University Medical Center/Maastricht University. The patients/participants provided their written informed consent to participate in this study.

## Author Contributions

MG: conceptualization, investigation, formal analysis, resources, writing – original draft, and writing – review & editing. AL: conceptualization, resources, writing – review & editing, and supervision. KK: methodology, writing – review & editing, and supervision. LG: investigation, resources, data curation, and writing – review & editing. JH: resources, writing – review & editing, supervision, project administration, and funding acquisition. KM: conceptualization, methodology, resources, writing – review & editing, and supervision. CM: conceptualization, methodology, investigation, formal analysis, resources, data curation, writing – original draft, writing – review & editing, visualization, supervision, project administration, and funding acquisition. All authors contributed to the article and approved the submitted version.

## Conflict of Interest

The authors declare that the research was conducted in the absence of any commercial or financial relationships that could be construed as a potential conflict of interest.

## Abbreviations

CoM: Center of Mass
BoS: Base of Support
METC: Medical Ethics Committee
MUMC+: Maastricht University Medical Center
Base: Baseline of the eleventh to second last step before each perturbation
Pre: The final step before each perturbation
Post1–8: The recovery steps following each perturbation (1–8).

## References

[B1] Berg W. P. Alessio H. M. Mills E. M. Tong C. (1997). Circumstances and consequences of falls in independent community-dwelling older adults. Age Ageing. 26, 261–268. 10.1093/ageing/26.4.261 9271288

[B2] Berry S. D. Miller R. R. (2008). Falls: epidemiology, pathophysiology, and relationship to fracture. Curr. Osteoporos. Rep. 6, 149–154. 10.1007/s11914-008-0026-4 19032925 PMC2793090

[B3] Bohm S. Mademli L. Mersmann F. Arampatzis A. (2015). Predictive and reactive locomotor adaptability in healthy elderly: a systematic review and meta-analysis. Sports Med. 45, 1759–1777. 10.1007/s40279-015-0413-9 26487633 PMC4656697

[B4] Deandrea S. Lucenteforte E. Bravi F. Foschi R. La Vecchia C. Negri E. (2010). Risk factors for falls in community-dwelling older people: a systematic reviews and meta-analysis. Epidemiology 21, 658–668. 10.1097/EDE.0b013e3181e89905 20585256

[B5] Dos Santos L. O. de Abreu D. C. C. Moraes R. (2018). Performance of faller and nonfaller older adults on a motor–motor interference task. J. Mot. Behav. 50, 1–14. 10.1080/00222895.2017.1341380 28854123

[B6] Geerse D. J. Roerdink M. Marinus J. Hilten van J. J. (2019). Walking adaptability for targeted fall-risk assessments. Gait Posture 70, 203–210. 10.1016/j.gaitpost.2019.02.013 30901621

[B7] Hausdorff J. M. Rios D. A. Edelberg H. K. (2001). Gait variability and fall risk in community-living older adults: a 1-year prospective study. Arch. Phys. Med. Rehabil. 82, 1050–1056. 10.1053/apmr.2001.24893 11494184

[B8] Hof A. L. Gazendam M. G. Sinke W. E. (2005). The condition for dynamic stability. J. Biomech. 38, 1–8. 10.1016/j.jbiomech.2004.03.025 15519333

[B9] Karamanidis K. Arampatzis A. (2007). Age-related degeneration in leg-extensor muscle-tendon units decreases recovery performance after a forward fall: compensation with running experience. Eur. J. Appl. Physiol. 99, 73–85. 10.1007/s00421-006-0318-2 17063362

[B10] Karamanidis K. Epro G. McCrum C. König M. (2020). Improving trip-and slip-resisting skills in older people: perturbation dose matters. Exerc. Sport Sci. Rev. 48, 40–47. 10.1249/JES.0000000000000210 31663865

[B11] Kiss R. Schedler S. Muehlbauer T. (2018). Associations between types of balance performance in healthy individuals across the lifespan: a systematic review and meta-analysis. Front Physiol. 9:1366. 10.3389/fphys.2018.01366 30323769 PMC6172339

[B12] Lamb S. E. Jorstad-Stein E. C. Hauer K. Becker C. (2005). Development of a common outcome data set for fall injury prevention trials: the prevention of falls network Europe consesus. J. Am. Geriatr. Soc. 53, 1618–1622. 10.1111/j.1532-5415.2005.53455.x 16137297

[B13] Lord S. R. S. C. Menz H. B. Close J. C. T. (2011). Falls in Older People: Risk Factors and Strategies for Prevention. New York, NY: Cambridge University Press.

[B14] McCrum C. (2020a). Fall prevention in community-dwelling older adults. N. Engl. J. Med. 382, 2579–2580. 10.1056/NEJMc2005662 32579827

[B15] McCrum C. (2020b). Falls History Questionnaire Material in English, German and Dutch. Available online at: https://osf.io/hmjef/ (accessed March 1, 2021).

[B16] McCrum C. Gerards M. H. G. Karamanidis K. Zijlstra W. Meijer K. (2017). A systematic review of gait perturbation paradigms for improving reactive stepping responses and falls risk among healthy older adults. Eur. Rev. Aging Phys. 14:3. 10.1186/s11556-017-0173-7 28270866 PMC5335723

[B17] McCrum C. Karamanidis K. Grevendonk L. Zijlstra W. Meijer K. (2020). Older adults demonstrate interlimb transfer of reactive gait adaptations to repeated unpredictable gait perturbations. Geroscience 42, 39–49. 10.1007/s11357-019-00130-x 31776885 PMC7031170

[B18] McCrum C. Karamanidis K. Willems P. Zijlstra W. Meijer K. (2018). Retention, savings and interlimb transfer of reactive gait adaptations in humans following unexpected perturbations. Commun. Biol. 1:230. 10.1038/s42003-018-0238-9 30564751 PMC6294781

[B19] McCrum C. Lucieer F. van de Berg R. Willems P. Perez Fornos A. Guinand N.. (2019a). The walking speed-dependency of gait variability in bilateral vestibulopathy and its association with clinical tests of vestibular function. Sci. Rep. 9:18392. 10.1038/s41598-019-54605-0 31804514 PMC6895118

[B20] McCrum C. Willems P. Karamanidis K. Meijer K. (2019b). Stability-normalised walking speed: a new approach for human gait perturbation research. J. Biomech. 87, 48–53. 10.1016/j.jbiomech.2019.02.016 30827703

[B21] Mortaza N. Abu Osman N. A. Mehdikhani N. (2014). Are the spatio-temporal parameters of gait capable of distinguishing a faller from a non-faller elderly? Eur. J. Phys. Rehabil. Med. 50, 677–691. 24831570

[B22] Pai Y. C. Bhatt T. Wang E. Espy D. Pavol M. J. (2010b). Inoculation against falls: rapid adaptation by young and older adults to slips during daily activities. Arch. Phys. Med. Rehabil. 91, 452–459. 10.1016/j.apmr.2009.10.032 20298839 PMC2842602

[B23] Pai Y. C. Wang E. Espy D. D. Bhatt T. (2010a). Adaptability to perturbation as a predictor of future falls: a preliminary prospective study. J. Geriatr. Phys. Ther. 33, 50–55. 10.1097/JPT.0b013e3181defbb1 20718383 PMC3483070

[B24] Pai Y. C. Yang F. Bhatt T. Wang E. (2014). Learning from laboratory-induced falling: long-term motor retention among older adults. Age 36:9640. 10.1007/s11357-014-9640-5 24668268 PMC4082608

[B25] Patla A. Frank J. Winter D. (1990). Assessment of balance control in the elderly: major issues. Physiother. Canada 42, 89–97. 10.3138/ptc.42.2.089

[B26] Petterson B. Nordin E. Ramnemark A. Lundin-Olsson L. (2020). Proposals for continued research to determine older adults' falls risk. J. Frailty Sarcopenia Falls 5, 89–91. 10.22540/JFSF-05-089 33283074 PMC7711736

[B27] Pijnappels M. Bobbert M. F. van Dieën J. H. (2005). Push-off reactions in recovery after tripping discriminate young subjects, older non-fallers and older fallers. Gait Posture 21, 388–394. 10.1016/j.gaitpost.2004.04.009 15886128

[B28] R Core Team. (2019). R: A Language and Environment for Statistical Computing. Available online at: https://www.R-project.org/ (accessed July 15, 2020).

[B29] Ringhof S. Stein T. (2018). Biomechanical assessment of dynamic balance: specificity of different balance tests. Hum. Mov. Sci. 58, 140–147. 10.1016/j.humov.2018.02.004 29438911

[B30] Süptitz F. Moreno Catala M. Bruggemann G. P. Karamanidis K. (2013). Dynamic stability control during perturbed walking can be assessed by a reduced kinematic model across the adult female lifespan. Hum. Mov. Sci. 32, 1404–1414. 10.1016/j.humov.2013.07.008 24071548

[B31] Woollacott M. H. Tang P. F. (1997). Balance control during walking in the older adult: research and its implications. Phys. Ther. 77, 646–660. 10.1093/ptj/77.6.646 9184689

